# Non-sterilized fermentation of high optically pure d-lactic acid by a genetically modified thermophilic *Bacillus coagulans* strain

**DOI:** 10.1186/s12934-017-0827-1

**Published:** 2017-11-25

**Authors:** Caili Zhang, Cheng Zhou, Nilnate Assavasirijinda, Bo Yu, Limin Wang, Yanhe Ma

**Affiliations:** 1 CAS Key Laboratory of Microbial Physiological and Metabolic Engineering, Institute of Microbiology, Chinese Academy of Sciences, Beijing, 100101 People’s Republic of China; 2 State Key Laboratory of Microbial Resources, Institute of Microbiology, Chinese Academy of Sciences, Beijing, 100101 People’s Republic of China; 3 Department of Biology, Faculty of Science, King Mongkut’s Institute of Technology Ladkrabang, Bangkok, 10520 Thailand

**Keywords:** *Bacillus coagulans*, Genetic engineering, d-Lactic acid, Optically pure, Non-sterilized fermentation

## Abstract

**Background:**

Optically pure d-lactic acid (≥ 99%) is an important precursor of polylactic acid. However, there are relatively few studies on d-lactic acid fermentation compared with the extensive investigation of l-lactic acid production. Most lactic acid producers are mesophilic organisms. Optically pure d-lactic acid produced at high temperature not only could reduce the costs of sterilization but also could inhibit the growth of other bacteria, such as l-lactic acid producers.

**Results:**

Thermophilic *Bacillus coagulans* is an excellent producer of l-lactic acid with capable of growing at 50 °C. In our previous study, the roles of two l-lactic acid dehydrogenases have been demonstrated in *B. coagulans* DSM1. In this study, the function of another annotated possible l-lactate dehydrogenase gene (*ldhL3*) was verified to be leucine dehydrogenase with an activity of 0.16 units (μmol/min) per mg protein. Furthermore, the activity of native d-lactate dehydrogenase was too low to support efficient d-lactic acid production, even under the control of strong promoter. Finally, an engineered *B. coagulans* D-DSM1 strain with the capacity for efficient production of d-lactic acid was constructed by deletion of two l-lactate dehydrogenases genes (*ldhL1* and *ldhL2*) and insertion of the d-lactate dehydrogenase gene (*LdldhD*) from *Lactobacillus delbrueckii* subsp*. bulgaricus* DSM 20081 at the position of *ldhL1*.

**Conclusions:**

This genetically engineered strain produced only d-lactic acid under non-sterilized condition, and finally 145 g/L of d-lactic acid was produced with an optical purity of 99.9% and a high yield of 0.98 g/g. This is the highest optically pure d-lactic acid titer produced by a thermophilic strain.

**Electronic supplementary material:**

The online version of this article (10.1186/s12934-017-0827-1) contains supplementary material, which is available to authorized users.

## Background

Technologies for the production of fuels and plastics from renewable biomass are currently being developed to prevent environmental pollution and global warming. Polylactic acid (PLA) is considered as an important bioplastic due to its superior thermal stability, mechanical performance, hydrolysis resistance, and excellent biodegradability [1, 2]. Optically pure l-lactic acid (≥ 99%) is the main precursor of PLA, and addition of optically pure d-lactic acid resulted in high melting point and good appearance. So, the production of d-lactic acid has received increasing focus recently. However, both yield and productivity of d-lactic acid are lower than those of l-lactic acid till now [3, 4]. Chemical synthesis of lactic acid leads to a racemic mixture and environmental pollution, and 70–90% of lactic acid in the world is produced by microbial fermentation in industry [5].

Compared with the extensive investigation of l-lactic acid production, there are relatively few studies on d-lactic acid fermentation [6]. Several strains have been reported to produce d-lactic acid naturally, such as *Lactobacillus* species, *Sporolactobacillus inulinus* [4, 6]. Lactic acid is generated from the conversion of pyruvate into l-lactic acid and d-lactic acid by l-lactate dehydrogenases (l-LDHs, encoded by *ldhL*) and d-lactate dehydrogenases (d-LDHs, encoded by *ldhD*), respectively [7]. Since d-LDHs is the key gene in d-lactic acid production, some studies reported to obtain engineered d-lactic acid producing strains with the deletion of the native *ldhL* gene [6]. Then, some metabolically engineered strains, including *Saccharomyces cerevisiae*, *L. plantarum*, *Corynebacterium glutamicum* and *Escherichia coli* were constructed to produce d-lactic acid [8]. However, all the above strains are mesophilic and the fermentation medium needs to be sterilized, which increases the process cost. Additionally, strains have to reach certain requirements to be used in efficient lactic acid production, such as low-cost nutrition needed, high yield, high optical purity of product, low/no oxygen requirement and short fermentation time [9].

Thermophilic *Bacillus coagulans* almost meet all the above requirements. They can withstand various harsh conditions, such as low pH values, high temperatures, and no aeration [6]. Moreover, *B. coagulans* can utilize a broad range of inexpensive carbon resources, and produce optically pure l-lactic acid at 50–55 °C which is expected to minimize contamination during fermentation in industrial scale [7, 10]. Nonsterile fermentation can omit the medium and fermentor sterilization process and largely reduce the production cost [6, 11]. Hence, *B. coagulans* is expected to be an excellent d-lactic acid producer for industrial uses [12]. Although there are some examples about l-lactic acid production by *Bacillus* sp. under non-sterilized conditions, no studies about using *B. coagulans* for optically pure d-lactic acid production under non-sterilized fermentation process are reported until now [10, 11].

In this study, *B. coagulans* DSM1, an optically pure l-lactic acid producer, was chosen for genetic engineering for d-lactic acid production. The key gene for l-lactic acid production was replaced with *LdhD* from *L. delbrueckii* subsp*. bulgaricus* DSM 20081. This genetically engineered strain produced high optical purity of d-lactic acid under non-sterilized condition.

## Methods

### Strains, plasmids and culture conditions

Strains and plasmids used in this study are listed in Table 1. The construction of *B. coagulans* DSM1ΔldhL1ΔldhL2, whose l-lactate dehydrogenase genes (*ldhL1* and *ldhL2*) have been knocked out, was described previously [13]. For genetic manipulation, the BC medium (50 g/L sucrose, 10 g/L yeast extract, 2 g/L (NH_4_)_2_HPO_4_, 3.5 g/L (NH_4_)_2_SO_4_, 10 g/L Bis–Tris, 3 mg/L CaCl_2_, 5 mg/L MgCl_2_, 20 μL trace element mixture) was used and the culture condition was 45 °C with 120 rpm. Filter sterilized trance element mixture contained 0.2 mg/L CoCl_2_·6H_2_O, 0.01 mg/L CuCl_2_·2H_2_O, 0.3 mg/L H_3_BO_3_, 0.03 mg/L Na_2_MoO_4_·2H_2_O, 0.02 mg/L NiSO_4_·6 H_2_O, 0.03 mg/L MnCl_2_·4H_2_O and 0.05 mg/L ZnCl_2_ [14]. For fermentation, the culture condition was 50 °C with 120 rpm in 513 medium (50 g/L glucose, 10 g/L yeast extract, 30 g/L CaCO_3_) [10]. Plasmids based on pMH77 were constructed in *Lactococcus lactis* MG1363, which was grown in GM17 medium (5 g/L soy peptone, 5 g/L tryptone, 2.5 g/L yeast extract, 5 g/L meat extract, 0.5 g/L ascorbic acid, 0.25 g/L MgSO_4_·7H_2_O, 5 g/L K_2_HPO_4_, 5 g/L glycerol, 10 g/L glucose). Plasmids based on pNW33N were constructed in *E. coli* TOP10 or BL21 (DE3), which was cultured in Luria–Bertani (LB) medium. When appropriate, 40 μg/mL kanamycin (Kan) and 25 μg/mL chloramphenicol (Cm) were added to *E. coli* culture, and 7 μg/mL Cm was added to *B. coagulans* and *L. lactis* culture.

**Table 1:** Strains and plasmids used in this study

Strains or plasmids	Features	Source
*Bacillus coagulans*		
DSM1	Wide type	DSMZ, Germany
DSM1Δ*ldh*L1Δ*ldh*L2	*ldh*L1 and *ldh*L2 null mutant	This study
d-DSM1	d-lactic acid producing strain	This study
DSM1Δ*ldh*L1Δ*ldh*L2–p*ldh*L1–*Bcldh*D	*B. coagulans* DSM1Δ*ldh*L1Δ*ldh*L2 with plasmid pNW33n–p*ldh*L1–*Bcldh*D	This study
DSM1Δ*ldh*L1Δ*ldh*L2–p*ldh*D–*Bcldh*D	*B. coagulans* DSM1Δ*ldh*L1Δ*ldh*L2 with plasmid pNW33n–p*ldh*D–*Bcldh*D	This study
DSM1Δ*ldh*L1Δ*ldh*L2–p*ldh*L1–Ld*ldh*D	*B. coagulans* DSM1Δ*ldh*L1Δ*ldh*L2 with plasmid pNW33n–p*ldh*L1–Ld*ldh*D	This study
DSM1Δ*ldh*L1Δ*ldh*L2–p*ldh*D–Ld*ldh*D	*B. coagulans* DSM1Δ*ldh*L1Δ*ldh*L2 with plasmid pNW33n–p*ldh*D–Ld*ldh*D	This study
*Lactococcus lactis* MG1363	Host for gene cloning	Laboratory preservation
*Escherichia coli* BL21(DE3)	Host for protein expression	Tiangen Co., China
*Escherichia coli* Top10	Host for gene cloning	Tiangen Co., China
pET-28a	Protein expression vector, Kan^R^	Merck Co., Germany
pGro7	Molecular chaperone, Cm^R^	TaKaRa Co., Ltd, China
pMH77	pSH71 replication containing temperature sensitive vector, Cm^R^	Laboratory preservation
pNW33n	*E. coli*-*Bacillus* shuttle vector, cloning vector, Cm^R^	BGSC, USA
pMH77–Ld*ldh*D	Ld*ldh*D insert vector, Cm^R^	This study
pNW33n–p*ldh*L1–*Bcldh*D	*Bcldh*D expression vector with *ldh*L1 promoter, Cm^R^	This study
pNW33n–p*ldh*D–*Bcldh*D	*Bcldh*D expression vector with *Bcldh*D promoter, Cm^R^	This study
pNW33n–p*ldh*L1–Ld*ldh*D	*ldh*L1 expression vector with Ld*ldh*D promoter, Cm^R^	This study
pNW33n–p*ldh*D–Ld*ldh*D	*ldh*D expression vector with Ld*ldh*D promoter, Cm^R^	This study

*Cm* ^*R*^ chloramphenicol resistant, *Kan* ^*R*^ kanamycin resistant

### Function verification of *ldhL3*

Gene *ldhL3* was cloned to pET-28a, and the recombinant plasmid was transformed into *E. coli* BL21 (DE3). To obtain the protein, isopropyl-β-d-1-thiogalactopyranoside (IPTG) was added to the culture with a final concentration of 0.5 mM when the OD_600_ value reached 0.6, and a continued culture was followed for 12 h at 16 °C. Cells were harvested (12,000×*g* for 10 min) and preserved at − 80 °C. After cell sonication, 6× His-Tagged Protein Purification Kit (CWBIO, China) was applied to obtain purified protein. If inclusion body formed, molecular chaperone (Takara, China) was used to help the protein expression.

For lactate dehydrogenase, the in vitro and in vivo enzymatic activities were studied as described previously [7]. For leucine dehydrogenase, reaction was carried out with 4.5 mM 4-methyl-2-oxopentanoate, 0.2 mM NADH and 1 mg purified enzyme in 0.1 M NH_4_Cl/NH_4_OH (pH = 9.5) at 45 °C. One unit of leucine dehydrogenase was defined as the amount of enzyme which catalyzed the consumption of 1 μmol of NADH per min under the standard assay conditions. Quantitative real-time (RT)-PCR was employed to determine the transcription levels of *ldhL3* in different fermentation periods. Total RNAs of *B. coagulans* DSM1ΔldhL1ΔldhL2 in logarithmic period, stationary period and decay period were isolated by using an E.Z.N.A bacterial RNA kit (Omega). With the primer pair of RTL3-F/R (Table 2), cDNA copies were synthesized with a FastQuant RT kit (with gDNase) (Tiangen, China) and amplified with SYBR Premix *Ex Taq* (TaKaRa, China). The threshold cycles (*C*_*T*_) for each PCR were analyzed according to the Ref. [13].

**Table 2:** Primers used in this study

Primers	Sequence (5′ → 3′)
L3-F	CCGGAATTCATGGAAATTTTTGATTATATGCG
L3-R	CCCAAGCTTTTACCGCGGAAGCCTTTTTTC
RTL3-F	TGAAAGAACCGAAGCACG
RTL3-R	CCGTTCTTCCGCCATCC
L1up-F	CCAGTACTCTGCAGAATTCGCTCCTTTCATTTGGTCAG
L1up-R	GTAAGCAAAAATTTTAGTCATATATAATCTTCCTCCCCATC
D-F	GATGGGGAGGAAGATTATATATGACTAAAATTTTTGCTTAC
D-R	GAAGCCCGGCCGGCACAAATGCTTAGCCAACCTTAACTGGAG
L1down-F	CTCCAGTTAAGGTTGGCTAAGCATTTGTGCCGGCCGGGCTTC
L1down-R	GCCGAAAATATGCACTCGAGGATCAACCGGGTCAGTGCAG
77-F	CTGCACTGACCCGGTTGATCCTCGAGTGCATATTTTCGGC
77-R	CTGACCAAATGAAAGGAGCGAATTCTGCAGAGTACTGG
pL1D-L1F	CGGGGTACCAGCCTCATCGCCGGTTTCC
pL1D-L1R	TAGGCAACAACTTTTCTCATATATAATCTTCCTCCCCATC
pL1D-DF	GATGGGGAGGAAGATTATATATGAGAAAAGTTGTTGCCTA
pL1D-DR	CCCAAGCTTTCATACTTTTATCTCCCACCTGCTC
pDD-F	CGGGGTACCGCATTCGTCTGAGTGGGCC
pDD-R	CCCAAGCTTTCATACTTTTATCTCCCACCTG
pL1LD-pL1F	GATTGTGAAATTGAATTCGAGCTCGGTACCAGCCTCATCGCCGGTTTCCCTCGC
pL1LD-LDR	GAAACAGCTATGACCATGATTACGCCAAGCTTTTAGCCAACCTTAACTGGAGTTTC
pDLD-pDF	CTGATTGTGAAATTGAATTCGAGCTCGGTACCGCATTCGTCTGAGTGGGCCAAGG
pDLD-pDR	GTAAGCAAAAATTTTAGTCATACGACAGCTTCCTTTCCATTC
pDLD-LDF	GAATGGAAAGGAAGCTGTCGTATGACTAAAATTTTTGCTTAC
pDLD-LDR	CTATGACCATGATTACGCCAAGCTTGCATGCCTGCAGTTAGCCAACCTTAACTGGAG

Restriction sites in the primer sequences are underlined

### Functional study of native *ldhD* in *B. coagulans* DSM1

The d-lactate dehydrogenase encoding gene from *B. coagulans* DSM1 (*BcldhD*) and *L. delbrueckii* subsp*. bulgaricus* DSM 20081 (*LdldhD*) were cloned to pNW33n under the control of the strong promoter of *ldhL1* promotor (P_*ldhL1*_, 1182 bp) and the native promoter of *BcldhD* (P_*BcldhD*_), respectively. In total, four recombinant plasmids in pNW33n, named as P_*ldhL1*_–*BcldhD*, P_*ldhL1*_–*LdldhD,* P_*BcldhD*_–*BcldhD* and P_*BcldhD*_–*LdldhD*, were constructed and transformed into *B. coagulans* DSM1ΔldhL1ΔldhL2. The concentration of d-lactic acid was measured.

### Gene insertion in *B. coagulans* DSM1ΔldhL1ΔldhL2

**Fig. 1:**
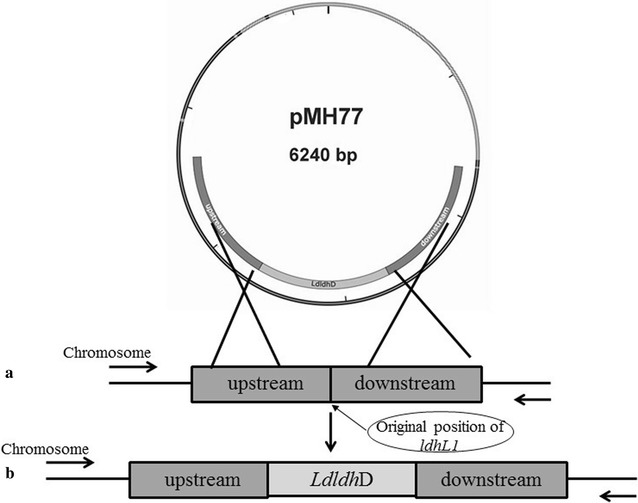
Insertion of *LdldhD* in the position of *ldhL1* gene of *B. coagulans* ΔldhL1ΔldhL2. **a** The upstream and downstream regions of *ldhL1* in the chromosome of *B. coagulans* ΔldhL1ΔldhL2; **b** The upstream and downstream regions of *ldhL1* in the chromosome of *B. coagulans* d-DSM1

Plasmid pMH77 was used as the primary vehicle to transfer *ldhD* from *L. delbrueckii* (*LdldhD*) to *B. coagulans* DSM1ΔldhL1ΔldhL2 in the original positon of *ldhL1* (Fig. 1). Primers used for gene insertion are listed in Table 2. Homologous arms of the upstream (1000 bp) and downstream (1000 bp) sequence of *ldhL1* were PCR amplified with primers L1up-F/R and L1down-F/R, respectively. *LdldhD* and pMH77 were amplified by primers DS-F/R and 77-F/R correspondingly. Then four fragments were connected by Clone Express^®^ MultiS One Step Cloning Kit (Vazyme Biotech Co., Ltd) resulting in plasmid pMH77–*LdldhD*.

Plasmid pMH77–*LdldhD* was firstly transformed into *L. lactis* MG1363 by electroporation. After verifying the correctness by sequencing, pMH77–*LdldhD* was transformed into *B. coagulans* DSM1ΔldhL1ΔldhL2 by electroporation. The transformants were cultured in BC medium with Cm at 45 °C for 6–10 h to mid-log phase and then the temperature was shifted to 60 °C for overnight [14]. A dilution series were plated on BC plates with Cm and incubated overnight at 60 °C. Colony PCR analysis was carried out for the first single crossover event. The positive clones were selected to be incubated in BC medium without Cm overnight at 45 °C. The obtained single colonies were firstly streaked onto BC plates with Cm and then without Cm. After incubation overnight at 45 °C, PCR analysis using the primers DS-For/Rev was conducted for the colonies which could grow on BC plates without Cm and could not grow on BC plates with Cm. The positive colonies were sequenced to confirm the gene insertion.

### Non-sterilized fermentation of d-lactic acid by *B. coagulans* D-DSM1

The seed cultures were prepared in 513 medium for 24 h and then inoculated with 10% (v/v) in the fermentation medium. The fermentation medium was maintained at approximately pH 5.5–6.0 with the addition of CaCO_3_. Fed-batch fermentation was carried out at 50 °C with 50 rpm in a 5-L bioreactor with 2 L fresh medium without sterilization. The initial glucose concentration was 50 g/L. When the glucose concentration decreased to lower than 20 g/L, glucose powder was added directly and repeatedly, leading to the result that the glucose concentration reached to approximately 50 g/L for 4 times.

### Analytical methods

Glucose consumption and lactate production were measured by HPLC (Agilent 1260 Series, Hewlett-Packard, Palo Alto, CA, USA) with an organic-acid column (MCI GEL CRS10W). The mobile phase was 6 mM H_2_SO_4_ and the flow rate was 0.5 mL/min. Temperature of the column was set to 55 °C. Both UV and differential detector were involved in the detection and the UV detection wavelength was 210 nm. The chiral and optical purity of d-lactic acid were analyzed using HPLC with a chiral column (MCI GEL CRS10W, Tokyo, Japan) [15]. Mobile phase was 2 mM CuSO_4_ at a flow rate of 0.5 mL/min. Column temperature was set to 25 °C and UV detection wavelength was 254 nm. The optical purity of d-lactic acid was calculated through the following formula: d-lactic acid optical purity = d-lactic acid/(d-lactic acid + l-lactic acid) × 100%. For the growth monitoring, A_600_ was measured by a 7200 Visible Spectrophotometer (UNICO, Shanghai, China).

## Results

### l-Lactate dehydrogenase gene (*ldhL3*) in *B. coagulans* DSM1ΔldhL1ΔldhL2

Three genes (*ldhL1*, *ldhL2* and *ldhL3*) related to l-lactic acid production were annotated in the genome of *B. coagulans* DSM1 (GenBank accession number CP009709). Gene *ldhL1* plays a major role in l-lactic acid production, and no L-lactic acid was detected in *B. coagulans* DSM1ΔldhL1ΔldhL2 [13]. Gene *ldhL3* is annotated as leucine dehydrogenase/l-lactate dehydrogenase. To verify the function of *ldhL3,* l-LDH3 (encoded by *ldhL3*) was expressed solubly in *E. coli* with the help of molecular chaperone *pGro7* [16]. The purified protein has the activity of leucine dehydrogenase with 0.16 units (μmol/min) per mg protein. RT-PCR and protein mass spectrometry studies showed that *ldhL3* expressed at both transcription level and protein level. However, neither d-lactate nor l-lactate was detected in the reaction product of purified l-LDH3 by HPLC. Although purified l-LDH3 could catalyzed the oxidation of NADH, only leucine dehydrogenase activity was detected. So, there is no l-LDH activity in *B. coagulans* DSM1ΔldhL1ΔldhL2, which makes the strain as a candidate for producing high optical purity of d-lactate.

### The activity of the native d-lactic acid dehydrogenase in DSM1ΔldhL1ΔldhL2

**Fig. 2:**
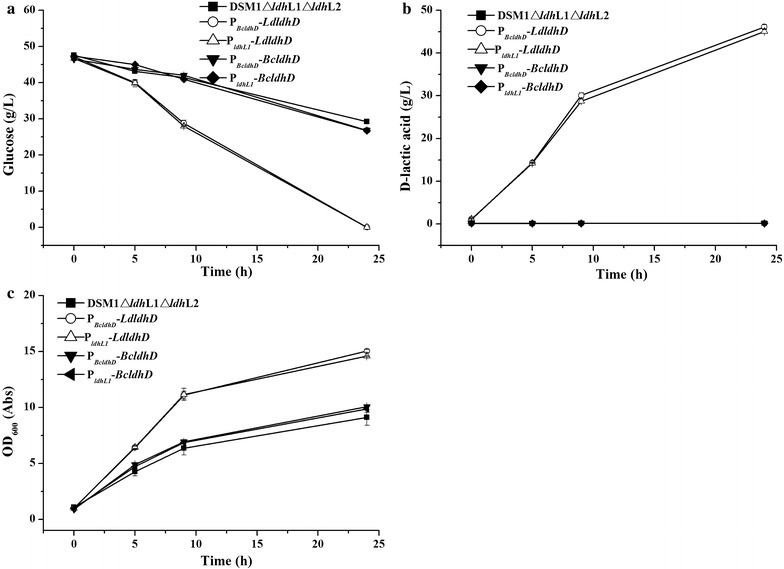
Fermentation profiles of *B. coagulans* DSM1ΔldhL1ΔldhL2 wild-type and mutant strains. **a** Glucose consumption; **b** d-lactic acid production; **c** A_600_ value. Each data point represents the average of three replicates, with the error bars representing the standard deviation

A D-LDH-encoding gene *BcldhD* was annotated in *B. coagulans* DSM1ΔldhL1ΔldhL2, and our previous study showed that the native d-LDH has an activity of 1.87 ± 0.08 U/mg in vitro [7, 13]. So, we firstly tried to detect the concentration of d-lactic acid produced by its native d-LDH in *B. coagulans* DSM1ΔldhL1ΔldhL2. However, only trace amount of d-lactic acid (0.26 g/L) was obtained (Additional file 1: Figure S1), and the growth of strain was poor when l-LDH genes deleted [13]. For *B. coagulans* DSM1, l-LDH deletion did not increase the expression or activity of native d-LDH. To trace the reason, a plasmid containing *ldhL1* promoter and native *ldhD* from *B. coagulans* DSM1 (*BcldhD*) was first constructed by using plasmid pNW33N (P_*ldhL1*_–*BcldhD*) and introduced into DSM1ΔldhL1ΔldhL2. Unfortunately, there was no difference in the concentrations of d-lactic acid between strain DSM1ΔldhL1ΔldhL2 carrying P_*ldhL1*_–*BcldhD* (0.15 ± 0.03 g/L) and strain DSM1ΔldhL1ΔldhL2 (0.16 ± 0.05 g/L) (Fig. 2). It seems that the enzyme activity of the native D-LHD in DSM1 was very low. *L. delbrueckii* subsp. *bulgaricus* DSM20081 is a d-lactic acid producer. The D-LDH (encoded by *LdldhD*) plays a central role in d-lactic acid production with a high catalytic efficiency (*k*_*cat*_) of 235.5 ± 1.6 s^−1^ [7]. To further investigate the mechanism, two strains carrying *LdldhD* under the control of *BcldhD* promoter (P_*Bcldh*_–*LdldhD*) and *ldhL1* promoter from DSM1 (P_*ldhL1*_–*LdldhD*) were constructed, respectively. Results showed that 46.14 ± 0.52 and 45.05 ± 0.47 g/L d-lactic acid were obtained, with a yield of 0.98 and 0.96 g/g, respectively (Fig. 2). Same glucose consumption rates and OD_600_ values were also observed in both strains.

### *B. coagulans* D-DSM1 construction by introducing *LdldhD*

Since the native d-lactate dehydrogenase could not support the efficient d-lactic acid production in the *ldhL* knockout strain, the *ldhD* gene from *L. delbrueckii* subsp. *bulgaricus* DSM20081 were introduced into *B. coagulans* DSM1ΔldhL1ΔldhL2 at the position of *ldhL1*, which is driven by the native promoter of *ldhL1* (Fig. 1). Using the primers of DS-For/Rev, a 3002 bp-band was detected by PCR amplification, which means that the gene *LdldhD* had been inserted into the positon of *ldhL1* in *B. coagulans* DSM1ΔldhL1ΔldhL2 (Additional file 1: Figure S2). The resultant strain *ldhL1*:: *LdldhD* was named as *B. coagulans* d-DSM1.

Strain *B. coagulans* d-DSM1 produced 48.65 g/L d-lactic acid with an initial glucose concentration of 50 and 117.96 g/L d-lactic acid with an initial glucose concentration of 120 g/L (Table 3). The optical purities were around 99.0–99.6%. To investigate the source of l-lactic acid in the broth, different yeast extraction concentration of 10, 5 and 1 g/L was added to the fermentation medium. At 0 h, 0.6 g/L l-lactic acid was detected in the medium with 10 g/L yeast extract, and the concentration was finally leveling out at 0.6 g/L during fermentation. The optical purity of d-lactate was 99.0, 99.5 and 100% respectively at 24 h. Therefore, it was concludes that the trace amount of l-lactic acid came from yeast extract and not from the endogenous metabolism of d-DSM1. Acetic acid (0.35 ± 0.02 g/L) and pyruvate (0.03 ± 0.00 g/L) were also found in the fermentation broth, and no ethanol, oxaloacetic acid and formic acid were detected (Additional file 1: Figure S3).

**Table 3:** d-Lactic acid fermentation with different initial glucose concentrations by *B. coagulans* d-DSM1

Glucose (g/L)	d-Lactic acid (g/L)	Yield (g/g)	Productivity (g/L/h)	Optical purity (%)
50	48.65 ± 0.43	0.97	2.03 ± 0.03	99.0
120	117.96 ± 0.73	0.98	1.97 ± 0.03	99.6

### Non-sterilized fed-batch fermentation of d-lactic acid

**Fig. 3:**
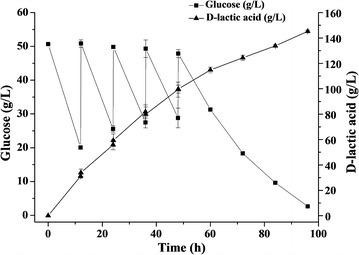
Non-sterilized fed-batch fermentation of *B. coagulans* d-DSM1. Each data point represents the average of three replicates, with the error bars representing the standard deviation

**Fig. 4:**
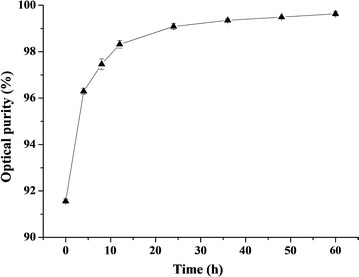
Optical purity of d-lactic acid in non-sterilized fed-batch fermentation of *B. coagulans* d-DSM1. Each data point represents the average of three replicates, with the error bars representing the standard deviation

Non-sterilized fermentation of d-lactic acid production was performed in a 5-L bioreactor with fed-batch strategy. The initial glucose concentration was 50 g/L. When residual glucose decreased to approximately 20 g/L, glucose was added to a concentration of 50 g/L. During the first 12 h, the concentration of glucose decreased rapidly from 50 to 20 g/L. The final concentration of d-lactic acid reached 145.23 ± 0.82 g/L with a yield of 0.98 g/g and a productivity of 1.51 ± 0.05 g/L/h (Fig. 3). Since yeast extract contained a small amount of l-lactic acid (0.4 g/L at 0 h), the optical purity of d-lactic acid was just 91.6% at 0 h and increased gradually to 99.2% at 24 h. Interestingly, the optical purity achieved about 100% at the end of fermentation (Fig. 4).

## Discussion

Poly-d-lactic acid is an important polymer because it improves the thermostability of poly-l-lactic acid through stereo complex formation. However, fermentation of d-lactic acid monomer has been little studied in comparison with l-lactic acid [17]. Till now, most d-lactic acid producing strains are mesophilic microorganisms, whose growth temperature is between 30 and 42 °C (Table 4) [6]. For example, Yamada et al. reported that 60 g/L d-lactic acid was obtained by genetically manipulated *S. cerevisiae* at 30 °C [18]. The main drawback of d-lactic acid production by mesophilic microorganisms is the contamination during fermentation, because most species of l-lactic acid producers display mesophilic properties. To overcome such technical shortcomings, strains with thermotolerant characteristics are highly desirable. Tashiro et al. isolated a strain named *L. delbrueckii* subsp. *lactis* QU 41, which could produce d-lactic acid at 49–55 °C [4]. *B. coagulans* strains typically produce l-lactic acid with low or undetectable level of d-lactic acid. Compared to the most of d-lactic acid producers, such as *L. bulgaricus*, *L. delbrueckii* and *L. plantarum*, *B. coagulans* does not need complex nutrients and require less cooling [19]. *B. coagulans* could growth under micro aerobic conditions, which do not need oxygen application and high-speed agitation during lactic acid production. So, *B. coagulans* is an promising host with robustness for d-lactic acid production.

**Table 4:** Comparison of d-lactic acid production by recombinants strains

Organism	Fermentation temperature (°C)	Fermentation mode	pH	d-Lactate concentration (g/L)	Total sugar addition	Yield (g/g)^a^	Productivity (g/L/h)^b^	Optical purity (%)	References
*Saccharomyces cerevisiae*	30	Batch	–	60	100 g/L (glucose)	0.65	2.8	99.9	[18]
*Pediococcus acidilactici*	42	SSF	–	97	25% (*w/w*, corn stover)	0.93	1.0	99.1	[24]
*Lactobacillus plantarum*	37	SSF	5.5–6.0	117	20% (w/v, brown rice)	0.93	0.8	99.6	[2]
*Saccharomyces cerevisiae*	30	Fed-batch	–	112	~190 g/L (glucose)	0.80	2.2	–	[25]
*Escherichia coli*	35	Batch	7.0	96	10% (*w/v*, sucrose)	0.93	2.6	> 99.5	[26]
*Sporolactobacillus inulinus*	42	Batch	5.0	93	100 g/L (glucose)	0.93	1.1	–	[27]
*Bacillus* sp.	54	Batch	6.5	28	50 g/L (sucrose)	0.96	–	99.5	[23]
*Bacillus coagulans*	50	Batch	5.0	90	110 g/L (glucose)	0.96	1.3	100	[12]
*Bacillus coagulans*	50	Batch	5.5–6.0	118	120 g/L (glucose)	0.98	2.0	99.6	This study
*Bacillus coagulans*	50	Fed-batch	5.5–6.0	145	150 g/L (glucose)	0.98	1.5	99.9	This study

*SSF* simultaneous saccharification and fermentation

^a^Yield (g/g) was calculated as the ratio of d-lactate produced (g) to consumed sugars (g)

^b^Productivity (g/L/h) was calculated as the ratio of d-lactate concentration (g/L) to the fermentation time (h)

The key enzymes of lactic acid production are lactate dehydrogenases. l-lactate dehydrogenases (l-LDHs) are responsible for l-lactic acid production and d-lactate dehydrogenases (d-LDHs) are responsible for d-isomer production [7]. So many genetic modifications of strains focused on encoding genes of above two enzymes [20–23]. Actually, a d-lactic acid producing strain *B. coagulans* QZ19 was constructed by deleting the native *ldhL* and *alsS* (acetolactate synthase encoding gene) to impede anaerobic growth. Combing the growth-based selection, glycerol dehydrogenase was found to have the ability of d-LDH. Finally, 90 g/L of optically pure d-lactic acid was produced after a long time metabolic evolution in batch fermentation [12]. Low d-lactic acid titer hinders the further use for this strain. *B. coagulans* DSM1 is a homo-fermentative l-lactic acid producer. Three l-lactate dehydrogenase (l-LDH) encoding genes (*ldhL1*, *ldhL2* and *ldhL3*) were discerned according to the whole-genome sequence of *B. coagulans* DSM1. The key roles of *ldhL1* and *ldhL2* have been systematically studied in our previous paper [13]. Both l-LDH1 (encoded by *ldhL1*, 5.27 ± 0.14 U/mg) and l-LDH2 (encoded by *ldhL2*, 4.53 ± 0.26 U/mg) were found to have catalytic activities in vitro. However, no transcription level was detected in *ldhL2* in vivo. Deletion of the *ldh*L2 gene revealed no difference in fermentation profile compared to the wild-type strain, while *ldh*L1 single deletion or *ldh*L1*ldh*L2 double deletion completely blocked l-lactic acid production. Therefore, the genes encoding l-LDH1 (*ldh*L1) was inferred to contribute to l-lactic acid synthesis in *B. coagulans* DSM1. Because lactate dehydrogenases drive the consumption of NADH, which is an important step in the metabolism and energy conversion of living cells, *ldhL1* deletion may provoke the increased expression of other NADH consumption enzymes, such as l-LDH2 [21]. So, *B. coagulans* DSM1ΔldhL1ΔldhL2 was used as a candidate in this study.

Because gene *ldhL3* is annotated as leucine dehydrogenase/l-lactate dehydrogenase, the enzymatic characterization of l-LDH3 (encoded by *ldhL3*) was firstly studied. l-LDH3 was confirmed to be leucine dehydrogenase. There is no l-LDHs in *B. coagulans* DSM1ΔldhL1ΔldhL2. Then we tried to use the native d-LDH encoding gene *BcldhD* from *B. coagulans* DSM1 to construct the d-lactic acid producer. However, only trace amount of d-lactic acid was produced in *B. coagulans* DSM1ΔldhL1ΔldhL2, and the growth of strain was poor when l-LDH encoding genes deleted (Additional file 1: Figure S1) [12]. To trace the reason for low d-lactic acid production, d-lactic acid productions using four recombinants, expressing different d-LDH encoding genes under the control of *BcldhD* promoter (P_*BcldhD*_) and *ldhL1* promoter (P_*ldhL1*_) respectively, were performed. The introduction of *BcldhD* under the control of P_*BcldhD*_ or P_*ldhL1*_ led to low production of d-lactic acid and low consumption of glucose. However, the introduction of a d-LDH encoding gene *LdldhD* from *L. delbrueckii* subsp. *bulgaricus* DSM20081 under the control of P_*BcldhD*_ or P_*ldhL1*_ resulted in high production of d-lactic acid and high consumption of glucose (Fig. 2), which suggested that the activity of native promoter of *BcldhD* functioned enough to drive the gene expression. Even when strong promoter (P_*ldhL1*_) was introduced to drive the high expression of *BcldhD*, the enzyme of d-LDH from strain DSM1 did not show sufficient activity. So, the activity of native d-LDH was too low to support the efficient d-lactic acid production.

*Lactobacillus delbrueckii* subsp. *bulgaricus* DSM20081 is a d-lactic acid producer. Its key enzyme d-LDH, encoded by *Ldldh*D, exhibited a high catalytic efficiency [7]. Most importantly, d-LDH from strain DSM20081 could work at the temperature at 50 °C, which is consistent with the growth temperature of *B. coagulans* (Additional file 1: Figure S4). Gene *LdldhD* from strain DSM20081 was inserted into the original location of *ldhL1* using the *ldhL1* promoter (Fig. 1). The obtained strain d-DSM1 produced 145 g/L d-lactic acid in fed-batch fermentation, with a yield of 0.98 g/g and an optical purity of 99.9% (Fig. 3), and 118 g/L d-lactic acid with a yield of 0.98 g/g was obtained in batch fermentation (Table 3). Attempts have been made to produce d-lactic acid using Lactic Acid Bacteria and *E. coli* [2, 26]. Some acid-tolerant strains, such as *S. cerevisiae*, *P. acidilactici*, *S. inulinus*, were also engineered to produce d-lactic acid [18, 24, 25, 27]. However, low d-lactic acid concentration was obtained under non-neutralized condition [25]. Actually, most strains are mesophilic (Table 4). A d-lactic acid producing strain was constructed based on a thermophilic Bacillus strain and 28 g/L d-lactic acid with an optical purity of 99.5% was obtained [23]. For thermophilic organism, the maximal yield of d-lactic acid was 90 g/L in batch fermentation [12]. In this study, besides metabolic engineering, also process optimization was carried out to obtain a thermophilic d-lactic-acid-producing strain. Compared with the existing d-lactic acid producing strains, strain d-DSM1 obtained in this study is more suitable for industry uses with high d-lactic acid titer (145 g/L) and high optical purity (99.9%). Until now, it is the highest d-lactic acid titer produced by thermo-tolerant strains (Table 4).

In conclusion, this present strain d-DSM1, which expresses *Ldldh*D from *L. delbrueckii* subsp. *bulgaricus* DSM20081 under the control of the native *ldhL1* promoter, produced d-lactic acid with high yield (0.98 g/g) and high optical purity (99.9%). Furthermore, non-sterilized fermentation was conducted which would significantly lower the fermentation costs and nutrition loss, and thus increase the economy of the fermentation process. *B. coagulans* d-DSM1 could be a promising d-lactic acid producer in industrial settings.

## Additional file

**Additional file 1.** Additional figures.
