# Supplementary material for: Non-sterilized fermentation of high optically pure d-lactic acid by a genetically modified thermophilic Bacillus coagulans strain
Source: Microb Cell Fact. 2017 Nov 25;16:213. doi: 10.1186/s12934-017-0827-1 (PMC5702109; doi:10.1186/s12934-017-0827-1)
Supplement: Supplementary file 1 — Additional file 1. Additional figures. [file 12934_2017_827_MOESM1_ESM.doc]

**Additional information for**

**Non-sterilized fermentation of high optically pure D-lactic acid by a genetically modified thermophilic *Bacillus coagulans* strain**

Caili Zhang^1†^, Cheng Zhou^2†^, Nilnate Assavasirijinda^3^, Bo Yu^1^, Limin Wang^1,*^, Yanhe Ma^2^

^1^ *CAS Key Laboratory of Microbial Physiological and Metabolic Engineering, Institute of Microbiology, Chinese Academy of Sciences, Beijing 100101, PR China*

^2^ *State Key Laboratory of Microbial Resources, Institute of Microbiology, Chinese Academy of Sciences, Beijing 100101, PR China*

^3^ *Department of Biology, Faculty of Science, King Mongkut's Institute of Technology Ladkrabang, Bangkok 10520, Thailand*

† Contributed equally to this study.

* Corresponding author.

E-mail: [wanglimin@im.ac.cn](mailto:wanglimin@im.ac.cn)

Phone/Fax: +86-10-64806132


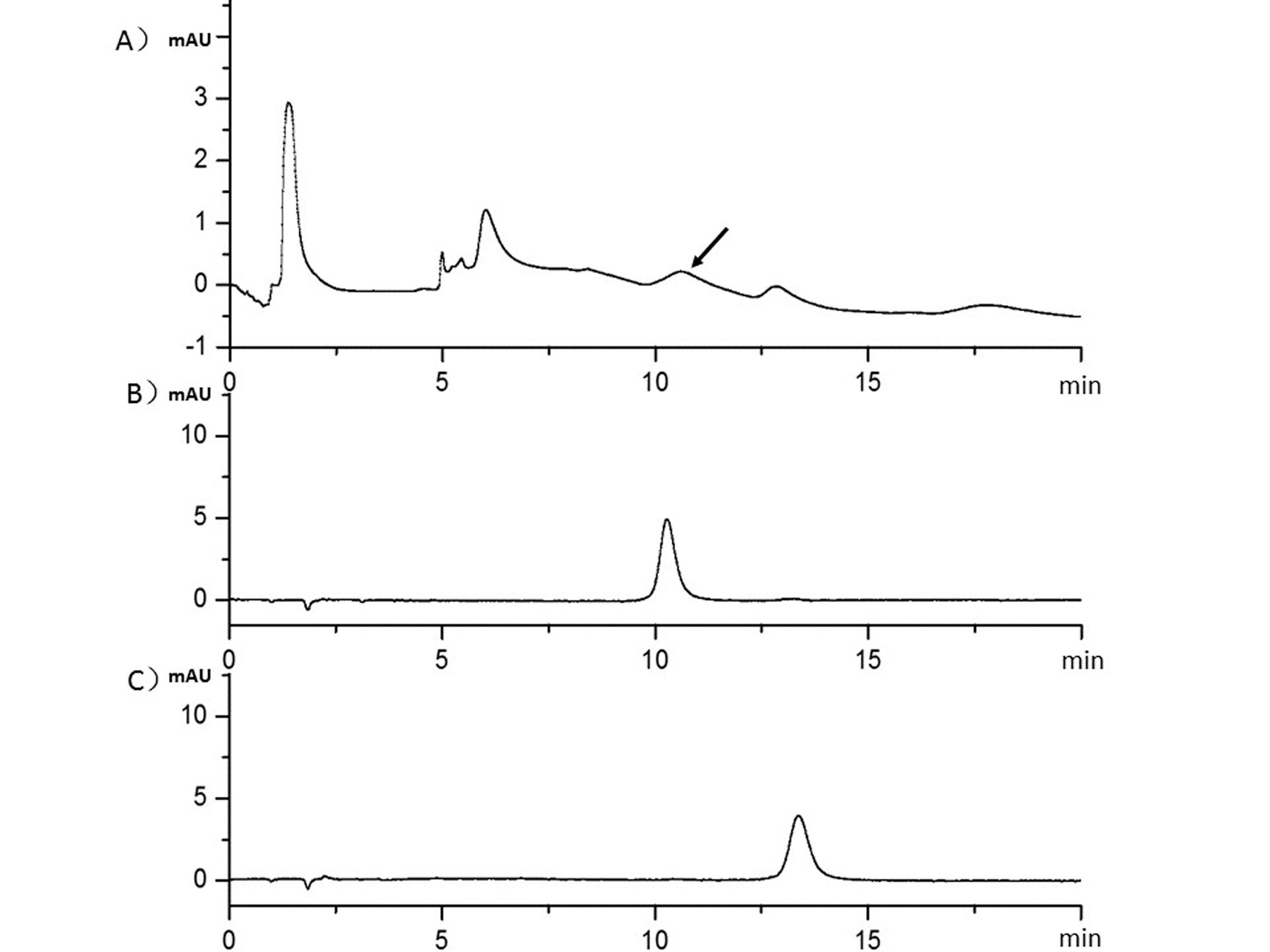


**Figure** **S1**. **HPLC detection of crude enzyme reaction**

A: Crude enzyme reaction (The arrow indicated the product peak of D-lactic acid); B: Standard of 0.1 mM D-lactic acid; C: Standard of 0.1 mM L-lactic acid


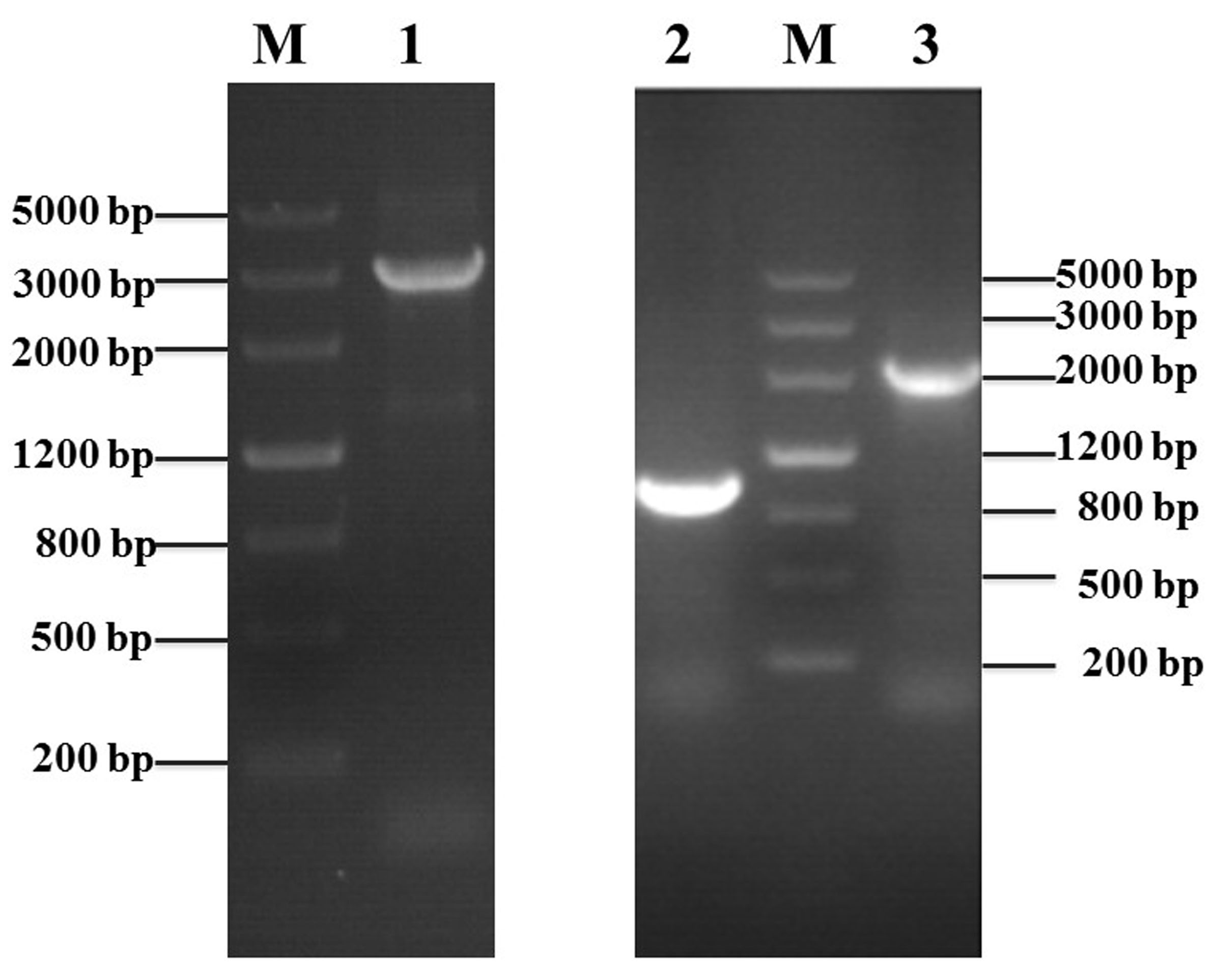


**Figure** **S2**. **PCR confirmation of *LdldhD* insertion into the positon of *ldhL1* in *B. coagulans* DSM1△ldhL1△ldhL2.**

Lane 1: *ldhL1* upstream (1000 bp)+ *LdldhD* (1002 bp)+ *ldhL1* downstream (1000 bp); Lane 2: *LdldhD* (1002 bp); Lane 3: P*_ldhL1_* (1182 bp)+ *LdldhD* (1002 bp).

**
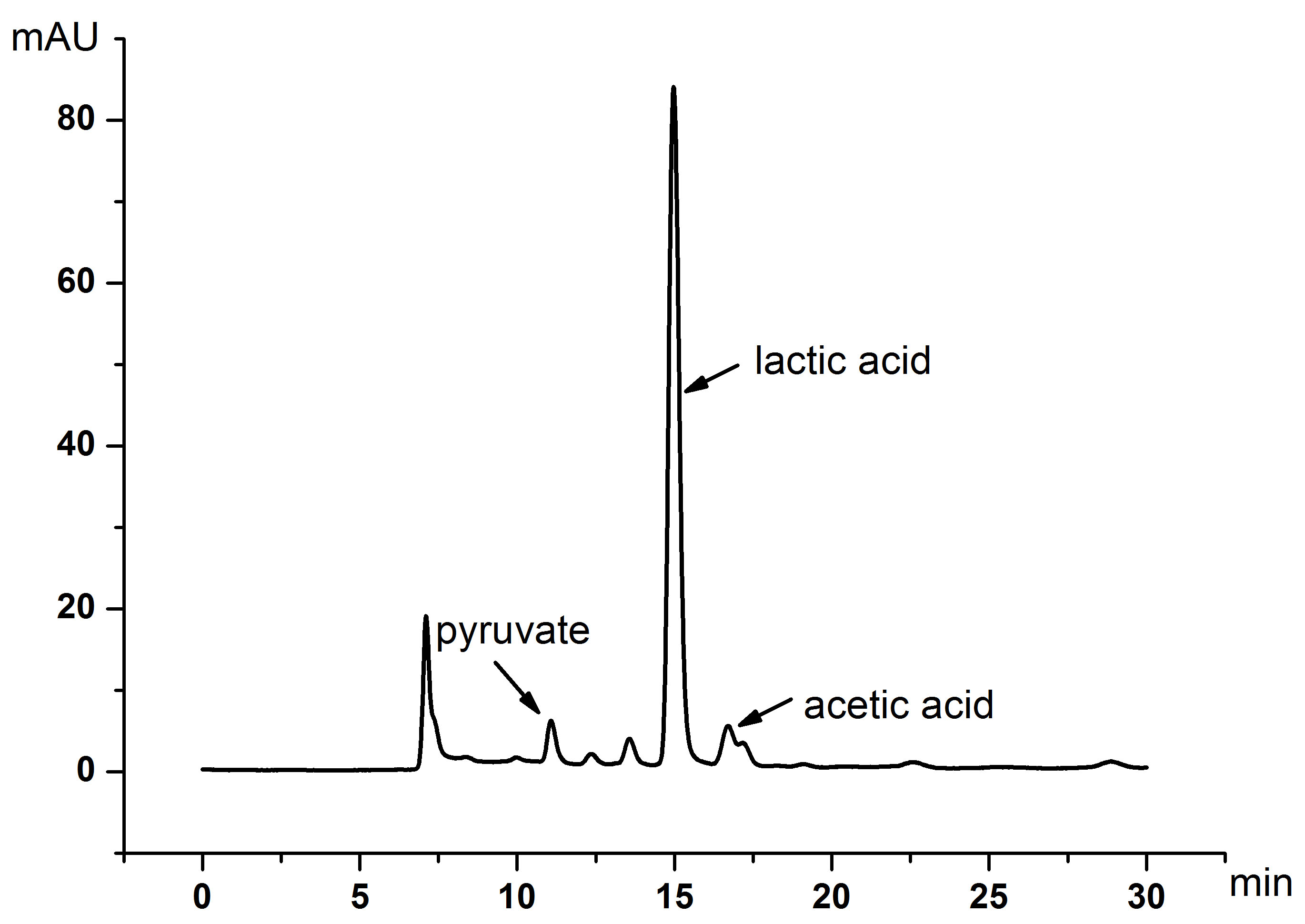
**

**Figure** **S3**. **HPLC analysis of fermentation products in *B. coagulans* D-DSM1.**


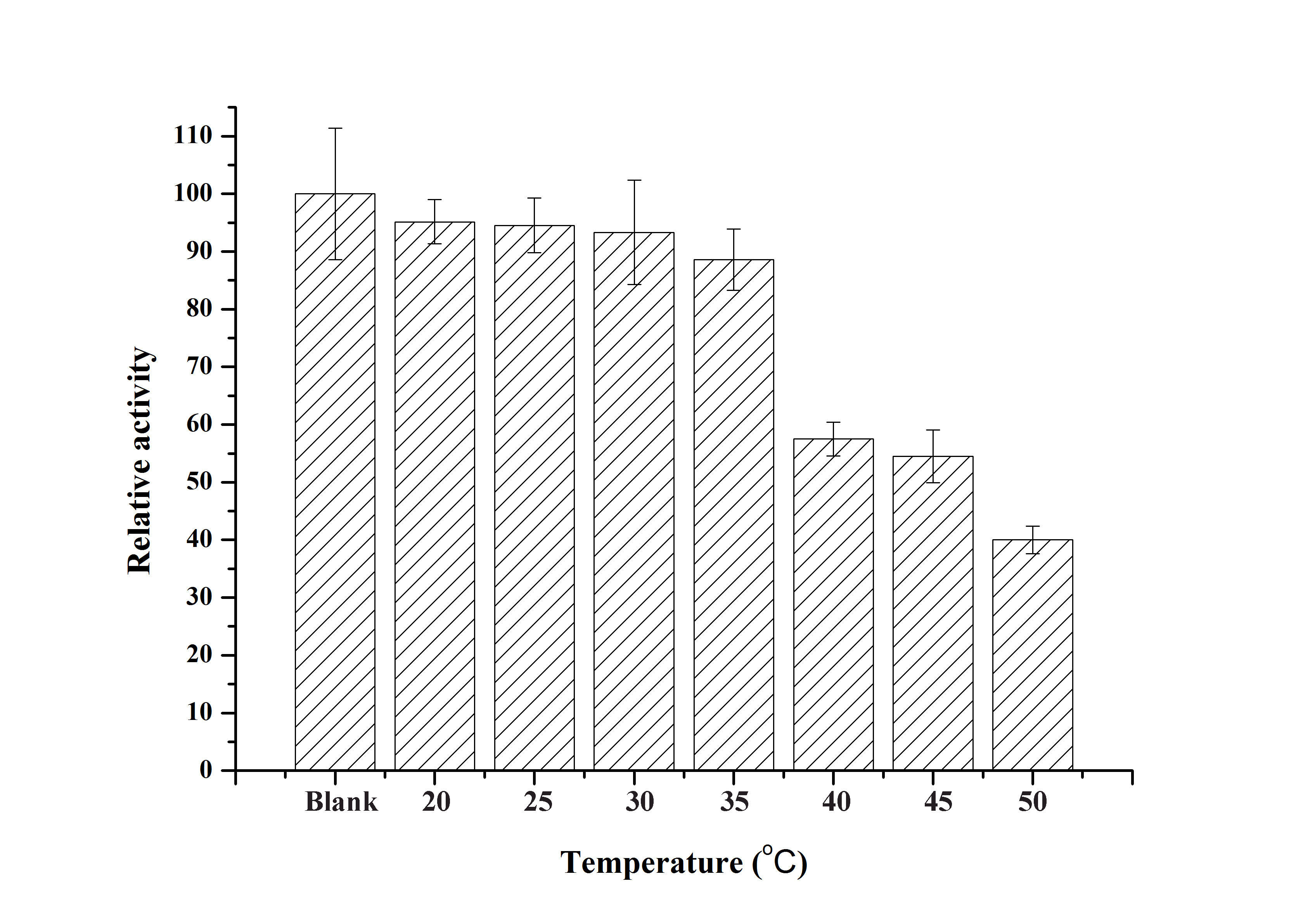


**Figure** **S4**. **The thermos-stability of D-LDH from *L. delbrueckii* subsp. *bulgaricus* DSM20081.**
